# Chylopericardium in a child with IgA nephropathy: a case report

**DOI:** 10.1186/s12887-018-1101-3

**Published:** 2018-04-05

**Authors:** Yu-lin Kang, Yun Cui, Ying Wu, Shen Hao, Xin-yu Kuang, Yu-cai Zhang, Wen-yan Huang, Guang-hua Zhu

**Affiliations:** 1 Department of Nephrology and Rheumatology, Shanghai Children’s Hospital, Shanghai Jiao Tong University, Shanghai, China; 2 Pediatric Intensive Care Unit, Shanghai Children’s Hospital, Shanghai Jiao Tong University, Shanghai, China

**Keywords:** Chylopericardium tamponade, IgA nephropathy, Pericardiocentesis, Pericardium drainage, Middle chain triglyceride

## Abstract

**Background:**

Chylopericardium effusion is characterized by the accumulation of milky effusion in the pericardium. It is often idiopathic but it can be secondary to trauma, chest radiation, tuberculosis and malignancy. If cardiac tamponade ensues, it becomes life-threatening. Herein we describe chylopericardium tamponade in a child with IgA nephropathy. To the best of our knowledge, this is the first reported case of chylopericardium tamponade in IgA nephropathy.

**Case presentation:**

A 6 years old boy with IgA nephropathy presented with dyspnea, orthopnea, pretibial pitting edema, ascites and fever. Muffled heart sounds and hepatomegaly were also noted. Echocardiography and thoracic CT revealed that there was a large volume of hydropericardium. Moreover, the pericardial milky fluid by pericardiocentesis was analyzed and chylopericardium effusion was eventually confirmed. Pericardial drainage was continued and his diet was modified to low fat, rich MCT and high protein. Complete remission was achieved after 3 weeks of this combined treatment.

**Conclusion:**

Chylopericardial tamponade could be a rare and life-threatening complication of IgA nephropathy. Etiological analysis is critical for determining the therapeutic approach in patients with pericardial effusion.

## Background

Pericardial effusion is a rare complication in renal diseases and could lead to hemodynamic compromise. Patients may initially present with clinical tamponade, characterized by dyspnea, tachycardia, jugular venous distension or even shock, while others may be asymptomatic if pericardial fluid accumulation is small [1, 2]. Pericardial effusion may be idiopathic or secondary to underlying diseases, such as acute myocardial infarction, end stage renal disease and autoimmune diseases [3–5]. Prompt diagnosis is critical. However, chylopericardial effusions may be difficult to diagnose unless the pericardial fluid undergoes chemical analysis. It is recognized that chylopericardium effusion may result from trauma, infection or blockage of the lymphatics [6]. In this paper, to the best of our knowledge, we are presenting the first case of chylopericardial tamponade in a child with IgA nephropathy.

## Case presentation

A 6 years old boy with dyspnea, orthopnea, generalized pitting edema and fever, was admitted to the pediatric intensive care unit, Shanghai Children’s Hospital. Three years before this episode, he presented to our hospital with acute onset of edema, hypoalbuminemia, heavy proteinuria and hyperlipidemia. The diagnosis of IgA nephropathy (Grade II) was made by percutaneous renal biopsy and in accordance with Lee’s classification [7]. Initially he responded well to steroid therapy, but became steroid resistant after 2 years treatment. Immunosuppressive agents administered during this time period, included cyclophosphamide, mycophenolate mofetil and tacrolimus. He had no history of trauma, tuberculosis or radiation therapy.

**Fig. 1:**
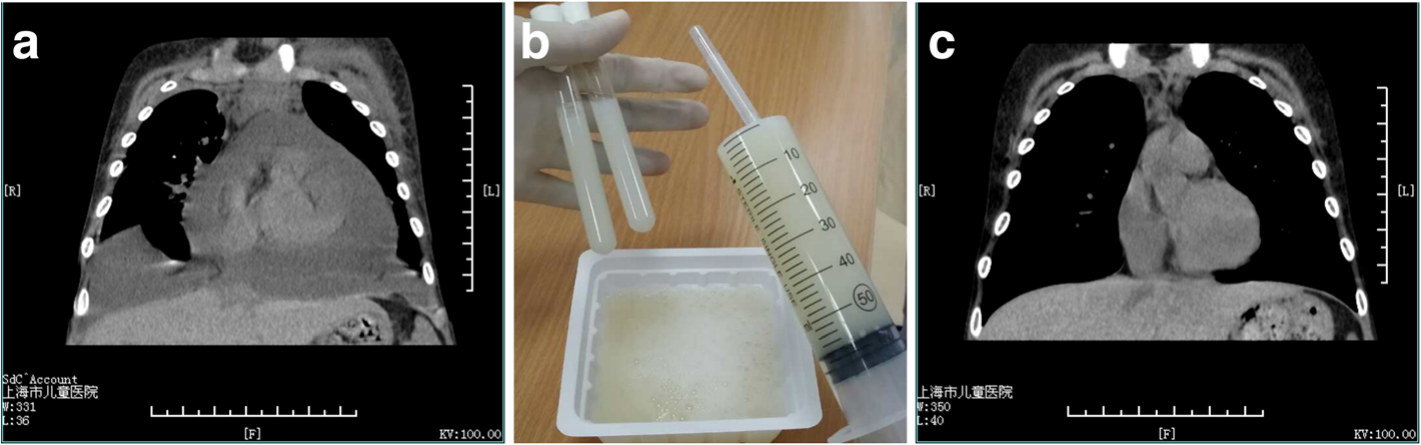
**a** Thoracic CT demonstrating massive pericardial and pleural effusion. **b** The milky pericardial effusion was obtained from the child with IgA nephropathy. Laboratory tests revealed that this milky pericardial fluid was saturated chylous effusion. **c** The thoracic CT demonstrating resolution of pericardial effusion after pericardial drainage and modified diet therapy

Physical examination revealed tachypnea, orthopnea, anasarca and ascites. The heart sounds were muffled and hepatomegaly was also noted. Blood pressure ranged from 90/60 mmHg (systolic/diastolic blood pressure) to 130/70 mmHg. Blood cell count showed that white blood cells (WBC) was 14.49 × 10^9^/L, neutrophils 79%, hemoglobin 11.5 g/dl, C-reactive protein 130 mg/L. Biochemistry analysis revealed total protein of 36 g/L, albumin 10 g/L, alanine aminotransferase (ALT) 10 U/L, aspartate aminotransferase (AST) 29 U/L, triglycerides 2.05 mmol/L, cholesterol 10.13 mmol/L. Serum electrolytes (Na^+^, K^+^, Ca^2+^ and Cl^−^) were normal. Heavy proteinuria and hematuria were found on urinalysis (urinary protein: creatinine ratio 30.38). Serum creatinine was normal and estimated glomerular filtration rate (eGFR, calculated with Schwartz formula) was 147 ml/min/1.73m^2^. Blood and urine cultures were sterile. T-spot for tuberculosis was negative. There was no ultrasound evidence of thrombosis in the superior vena cava or subclavian vein. Ultrasound also demonstrated that both kidneys were enlarged with a loss of cortico-medullary differentiation. Thoracic computed tomography (CT) found no evidence of congenital malformation or malignancies. Echocardiography revealed pericardial fluid of 4.6 cm at maximal thickness, suggesting a large volume hydropericardial effusion. The massive pericardial and pleural effusions were additionally confirmed by thoracic CT (Fig. 1a). Notably, milky fluid was obtained from the pericardial space by pericardiocentesis (Fig. 1b). Chyle test was positive. Cell counts and biochemistry in the pericardial effusion revealed WBC 405 × 10^6^/L, lymphocytes count 92%, red blood cells 63 × 10^6^/L, AST 8 U/L, lactate dehydrogenase (LDH) 58 U/L, Glucose 7.22 mmol/L, total protein 7 g/L, albumin 4 g/L, adenylate deaminase (ADH) 2.6 U/L, triglycerides 2.55 mmol/L, cholesterol 0.79 mmol/L. These findings confirmed the diagnosis of chylopericardial effusion.

Continuous renal replacement (CRRT) therapy was performed to alleviate the fluid overload. Meanwhile, pericardial drainage was performed and the diet was modified to low fat but rich in middle chain triglycerides and high protein. No side effects were encountered with this diet modification. The symptoms of cardiac tamponade subsided promptly, while the edema receded gradually over the subsequent 2 weeks. The indwelling pericardial catheter was removed when no fluid was drained after 3 weeks treatment and as shown in Fig. 1c, the chylopericardial effusion was removed successfully. Pericardial effusion has not recurred at 1 year of follow up, on follow up echocardiography.

## Discussion and conclusions

Chylopericardium is characterized by a milky pericardial effusion, which is saturated with chyle and triglyceride. Clinical manifestations vary from asymptomatic to life threatening pericardial tamponade, depending on the extent of the effusion [8]. Our case highlights the importance of early diagnosis and treatment, as such patients can be easily misdiagnosed. Thoracic and pericardium effusion are seldom suspected to be chylous fluid in nephrotic syndrome unless the fluid sample is specifically analyzed for chyle and triglycerides. Thus, the awareness of chylopericardial tamponade needs to be heightened in renal or rheumatology diseases.

Chylopericardium can be idiopathic or secondary to trauma, tuberculosis, radiation, mediastinal tumor, venous thrombosis and congenital anomalies of the lymphatic duct [9]. Therefore, a history of blunt injury or thoracic operation needs to be reviewed. Neoplasm, thrombosis and congenital malformations should be looked for using CT scan, magnetic resonance imaging (MRI) or ultrasound. Tuberculosis can be excluded by the T-spot assay. Furthermore, lymphangiography and lymphoscintigraphy may be employed to exclude anatomic abnormities of thoracic lymphatic system [10, 11]. Additionally, it should be recognized that thrombosis and tuberculosis are two common complications seen in nephrotic syndrome and other glomerular diseases with nephrotic range proteinuria. As a result, chylopericardial effusion may occur if the integrity of the lymphatic duct was damaged by such conditions. However, no evidence of any of these causes was present in our case. The diagnosis of idiopathic chylopericardium may be made if no underlying cause is found. It has been reported that idiopathic chylopericardial effusion affects both genders equally and exists in different age groups [12, 13]. The etiology remains unclear, but it may due to elevated intrathoracic lymphatic pressure, increased permeability of lymphatic vessel walls, or valvular incompetence and ruptured lymphatic valves [6]. The most common manifestations of patients with idiopathic chylopericardial infusion range from absence of symptoms to cough, dyspnea and fatigue. Table 1 details clinical presentations of cases reported in the literature. Our case presented with life threatening dyspnea and orthopnea. Thus, chylopericardial effusion should be suspected, if patients with IgA nephropathy have hydropericardium, given the potential for life threatening complications despite its rarity.

**Table 1:** Clinical features of chylopericardium in children

Reference	Age	Gender	Symptoms and signs	Etiology	Treatment	Duration	Prognosis
Suri et al. [16]	4Y	Female	Orthopnea, anasarca, tachycardia, tachypnea, pulsusparadoxus, engorged neck vein, muffled heart sound	Superior vena cava thrombosis	Heparin infusion and oral warfarin; Modified diet with low fat but rich in MCT; Pericardial drainage.	15 days	No recurrence
Lope-castilla et al. [17]	2 M	Male	Dyspnea and anorexia	Idiopathic	Pericardiocentesis; Pericardial drainage and low-fat total parenteral nutrition enriched with MCT	36 days	No recurrence
Tan et al. [18]	5Y	Male	Cough and dyspnea, distant heart sounds.	Idiopathic	Ligation of the thoracic duct and the creation of a pericardial window	1 week	N/A
Rivera-Beltran et al. [15]	16Y	Male	Intermittent chest pain and dizziness	Idiopathic	Pericardiocentesis; MCT-rich diet; Octreotide; transabdominal ligation of the thoracic duct with pericardial-peritoneal shunting	Rapid recovery	No recurrence
Musemeche et al. [19]	12Y	Male	Orthopnea, distant heart tones	Lymphangiectasia	Total parenteral nutrition; Right thoracotomy with ligation of the thoracic duct	Rapid recovery	No recurrence
Attias et al. [20]	13Y	N/A	Chest pain and fatigue	Idiopathic	Ligation of thoracic duct and partial pericardectomy; MCT-rich diet	Rapid recovery	No recurrence

*Abbreviations*: *N/A* not available, *Y* year, *M* Month

Regarding therapeutic approaches, the most important step is to relieve the symptoms by urgent pericardiocentesis or pericardial drainage, if a life-threatening situation exists [14]. Drainage should not be terminated until the effusion recedes to a very small amount, as it may be helpful for self-repair of the damaged lymphatic tubule. In our case, it took 3 weeks for pericardial drainage to cease suggesting a relatively long duration for self-recovery. Meanwhile, some studies reported other surgical procedures such as ligature and excision of thoracic duct combined with partial pericardiectomy were necessary if pericardial effusion is recurrent [15]. Table 1 summarizes our review of the literature and details different treatments and outcomes. It also has been reported that administration of octreotide is effective in reducing intestinal absorption of fats [13].Most importantly it is necessary to remove any underlying causes for chylopericardial effusion. For instance, if thrombosis is the primary cause of chylopericardial effusion, thrombolytic therapy must be considered. In addition, conservative therapies are also required, including low fat diet based on middle chain triglycerides (MCT) or total parenteral nutrition. In our study, the combined therapy of pericardial drainage and modified diet with low fat but rich in MCT and high protein brought about complete remission. Prognosis is good if patients receive timely surgical intervention and medical management.

Chylopericardial effusion should be suspected in patients with IgA nephropathy, when signs of tamponade are present. It is critical to exclude underlying causes. Surgical procedures for draining chylous fluid and medical therapy such as a modified diet are effective therapeutic approaches. To date, the challenging issue in chylopericardium is identification of underlying causes. Hopefully, less invasive approaches will be developed in the near future. Additionally, long-term follow up is important to obtain data on the outcome and occurrence of this rare complication of common renal diseases such as IgA nephropathy.
